# Synergistic Effects of New Curcumin Analog (PAC) and Cisplatin on Oral Cancer Therapy

**DOI:** 10.3390/cimb45060319

**Published:** 2023-06-08

**Authors:** Abdelhabib Semlali, Sarra Beji, Ikram Ajala, Mohammed Al-Zharani, Mahmoud Rouabhia

**Affiliations:** 1 Groupe de Recherche en Écologie Buccale, Faculté de Médecine Dentaire, Université Laval, Québec, QC G1V0A6, Canada; sarra.beji.1@ulaval.ca (S.B.); ikram.ajala@etudiant-fst.utm.tn (I.A.); mahmoud.rouabhia@fmd.ulaval.ca (M.R.); 2 Biology Department, College of Science, Imam Mohammad Ibn Saud Islamic University (IMSIU), Riyadh 11623, Saudi Arabia; mmyalzahrani@imamu.edu.sa

**Keywords:** oral cancer, chemotherapy, cisplatin, PAC, proliferation, apoptosis

## Abstract

Oral cancer has traditionally been treated with surgery, radiotherapy, chemotherapy, or a combination of these therapies. Although cisplatin, a chemotherapy drug, can effectively kill oral cancer cells by forming DNA adducts, its clinical use is limited due to adverse effects and chemo-resistance. Therefore, there is a need to develop new, targeted anticancer drugs to complement chemotherapy, allowing for reduced cisplatin doses and minimizing adverse effects. Recent studies have shown that 3,5-Bis (4-hydroxy-3-methoxybenzylidene)-N-methyl-4-piperidine (PAC), a new curcumin analog, possesses anticancer properties and could be considered a complementary or alternative therapy. In this study, we aimed to assess the potential complementary effects of PAC in combination with cisplatin for treating oral cancer. We conducted experiments using oral cancer cell lines (Ca9-22) treated with different concentrations of cisplatin (ranging from 0.1 μM to 1 μM), either alone or in conjunction with PAC (2.5 and 5 μM). Cell growth was measured using the MTT assay, while cell cytotoxicity was evaluated using an LDH assay. Propidium iodide and annexin V staining were employed to examine the impact on cell apoptosis. Flow cytometry was used to investigate the effects of the PAC/cisplatin combination on cancer cell autophagy, oxidative stress, and DNA damage. Additionally, a Western Blot analysis was performed to assess the influence of this combination on pro-carcinogenic proteins involved in various signaling pathways. The results demonstrated that PAC enhanced the efficacy of cisplatin in a dose-dependent manner, leading to a significant inhibition of oral cancer cell proliferation. Importantly, treatment with PAC (5 μM) alongside different concentrations of cisplatin reduced the IC50 of cisplatin tenfold. Combining these two agents increased apoptosis by further inducing caspase activity. In addition, the concomitant use of PAC and cisplatin enhances oral cancer cell autophagy, ROS, and MitoSOX production. However, combined PAC with cisplatin inhibits the mitochondrial membrane potential (ΔΨm), which is a marker for cell viability. Finally, this combination further enhances the inhibition of oral cancer cell migration via the inhibition of epithelial-to-mesenchymal transition genes, such as E-cadherin. We demonstrated that the combination of PAC and cisplatin markedly enhanced oral cancer cell death by inducing apoptosis, autophagy, and oxidative stress. The data presented indicate that PAC has the potential to serve as a powerful complementary agent to cisplatin in the treatment of gingival squamous cell carcinomas.

## 1. Introduction

Over the last few decades, the common treatment of oral cancer has relied almost exclusively on tumor resection with or without chemotherapy and radiotherapy. Cisplatin and other platinum-based drugs were considered the most common and effective chemotherapy drugs used for treating oral cancer [1,2,3]. Cisplatin applies anticancer activity through different mechanisms, including the generation of DNA lesions and cell apoptosis [4,5]. Oral cancer treatment with this product has been shown to be very effective for several years, but it has many adverse effects [6,7]. These side effects, called late effects, appear a few months to years after chemotherapy; they can damage healthy cells while destroying cancer cells. Frequent side effects of cancer therapy include reduced blood immune cells, diarrhea, fatigue, pain, hair loss, hot flashes, and psychological stress. These side effects significantly hinder the efficacy of cancer treatment. To address this challenge, it is necessary to develop a novel class of targeted and potent chemotherapeutic drugs that can be used as alternative or complementary treatments. This approach would allow for reduced cisplatin doses, thus minimizing adverse effects and improving patient outcomes.

For this purpose, plant compounds and their analogs are being explored. As a matter of fact, natural products derived from a diversity of sources can stimulate and invigorate numerous physiological pathways, which can be beneficial in controlling many diseases, such as cancer. Thus far, over half of medications have been developed from natural compounds, including 75% of anticancer drugs [8,9,10].

In recent years, herbal medicine has gained considerable importance and has proven to be a promising remedy for cisplatin-induced toxicity. It has been reported that natural products and their derivates help with the chemo-sensitization of cisplatin-resistant cells by either up or downregulating specific signaling pathways that trigger apoptosis [11,12]. Curcumin, a turmeric compound isolated from *Curcuma longa*, commonly used as a spice, is well known for multiple properties with great applications in many chronic diseases attributed to its biological activities as antioxidant, anti-inflammatory, antimicrobial, and anticancer drugs [13]. However, it presents many limitations that hinder its clinical application (low bioavailability and color, etc.). Many curcumin analogs (EF24 and PAC) have been recently synthesized to improve these limitations and have anticancer properties. EF24 was shown to be bioactive against cancer cells and was suggested as a potential complementary chemotherapeutic drug to cisplatin [14]. Recently, another curcumin analog named HO-3867 was also reported to be an interesting antioxidant appendage that drastically inhibits cancer cell growth by inducing apoptosis [15]. PAC, a novel curcumin analog, is more stable in PBS and circulating blood [16], and is five times more efficient than curcumin in inducing apoptosis cancer cells [17]; it has recently been reported for its selective role in suppressing many growth tumors [14,16,17,18,19]. Our previous data with PAC (3,5-Bis (4-hydroxy-3-methoxybenzylidene)-N-methyl-4-piperidine), a newly synthesized curcumin analog, showed that this molecule presents a high potential therapeutic target for oral cancer by altering pro-survival signaling pathways, activating apoptosis autophagy, and inhibiting oxidative stress [19]. The effectiveness of PAC on breast and colon cancer was also documented [16,17,18]. However, until now, no data on its synergistic potential with cisplatin in treating oral cancer have been reported. It is well known that the cell death process is often mediated by many intrinsic and extrinsic signaling pathways, triggered mostly by DNA damage, autophagy, and cellular stress. These pathways play key roles in the pathogenesis and cancer progression [20,21]. Targeting the mechanisms of apoptosis, autophagy, and oxidative stress is a widely attractive aim in current cancer therapy. It has gained an important place in current research targeting the discovery of new and more effective anticancer drugs. Therefore, our objective was to study the effects of PAC in combination with cisplatin on the proliferation, apoptosis, autophagy, oxidative stress, and DNA damage of oral cancer cells.

## 2. Methods and Materials

### 2.1. Cell Culture

The Ca9-22 gingival epithelial cancer cell line was originally sourced from RIKEN BioResource Research Center in Tsukuba, Japan. The cells were cultured in RPMI-1640 medium (Gibco, Waltham, MA, USA), supplemented with L-glutamine, 5% fetal bovine serum (FBS), and 1% antibiotics (Thermo Fisher Scientific, Burlington, ON, Canada). The culture was maintained at 37 °C in a humidified atmosphere containing 5% CO_2_. The growth medium was refreshed every two days until the cells reached 80% confluence. The PAC used in the study was obtained from Dr. Ibrahim Al-Jammaz’s laboratory at King Faisal Specialist Hospital and Research Center in Riyadh, Saudi Arabia.

### 2.2. Viability Assay

Cells were exposed or not to various concentrations of PAC (1, 2.5, 5, and 10 μM) with and without cisplatin (Sigma Aldrich, Oakville, ON, Canada) used at (0.01, 0.1, 0.5, 0.8, and 1 nM) for 24 h and were then used to assess the cell growth by means of MTT (Sigma Aldrich, Oakville, ON, Canada) colorimetric assay, as described previously [19,22]. In brief, the cultures were supplemented with 10% MTT (5 mg/mL) and incubated at 37 °C for 3 h. After the incubation period, the resulting formazan crystals were dissolved in isopropanol −0.04 H HCL solution, and the plate was placed on an orbital shaker for 1 h at room temperature. The absorbance was read at 550 nm using an iMark microplate reader (Bio-Rad, Mississauga, ON, Canada). To calculate the percentage of cell viability, the absorbance of individual wells was divided by the mean absorbance of control wells from three independent experiments conducted in triplicate. Cell proliferation levels were determined using the following formula:

% of cell viability = [(OD 550 nm (treated cells) − OD (Blank))/(OD (control cells) − OD (Blank))] × 100.

### 2.3. Hoechst STAINING

The Ca9-22 cells (10^5^) were seeded on 12-well plates, cultured for 24 h, then treated with different concentrations of cisplatin (0, 0.01, 0.1, 0.5, 0.8, and 1 nM) with or without PAC (1, 2.5, 5, 10 μM) for 24 h. The cells were rinsed twice with cold PBS and then fixed with a solution of cold methanol and acetic acid (75%/25%) for a duration of 30 min. After fixation, the cells were washed with PBS and then suspended in a 1% Hoechst solution from sigma Aldrich. Subsequently, they were incubated in the dark for 15 min. Following another round of washing, the stained cells were examined and captured using a fluorescence microscope.

### 2.4. Clonogenic Assay

The Ca9-22 cells were seeded in the 6-well culture plates and exposed to various concentrations of PAC (2.5, and 5 μM) and cisplatin (0, 0.01, 0.1, 0.5, 0.8, and 1 nM) for two weeks. After the completion of the culture period, the culture medium was aspirated, and the adherent cells were washed with PBS. Subsequently, the cells were fixed with 4% methanol at room temperature for 10 min and then stained using 0.05% crystal violet. Following a 15 min incubation at room temperature, the dye was carefully removed, and the wells were thoroughly washed with PBS. The plates were allowed to dry, and colony formation was observed and captured using an inverted microscope. To quantify the colony formation, the crystal violet was solubilized with 200 µL of a 30% (*v*/*v*) acetic acid solution. The absorbance was measured at 570 nm using a Bio-Rad xMark reader in a 96-well plate (Bio-Rad, Mississauga, ON, Canada).

### 2.5. Cell Apoptosis Assay

Apoptosis was assessed using flow cytometry with annexin V-FITC/PI staining kit (BD Bioscience, Mississauga, ON, Canada) [19,22,23]. Cells were seeded in 6-well plates at a density of 3 × 105 cells/well and allowed to adhere overnight. They were then treated with different concentrations of cisplatin (ranging from 0.5 to 0.8 nM) in the presence or absence of PAC (2.5 and 5 μM) for 24 h. The selected concentrations were based on their proximity to the IC50 values. Following treatment, the cells were harvested, washed twice with cold PBS, and stained with annexin V-FITC and PI, as per the manufacturer’s instructions. The stained samples were analyzed using a flow cytometer equipped with FACS Diva software (version 6.1.3). The distribution of viable (annexin−/PI−), early apoptotic (annexin+/PI−), late apoptotic (annexin+/PI+), and necrotic (annexin−/PI+) cells was determined. Both early and late apoptotic cells were considered apoptotic cells, and the results were expressed as a percentage of the total cell population. The experiment was performed three times to ensure reliability and consistency.

### 2.6. Caspase Activity Assay

Caspase activities were evaluated using a Caspase detection kit (TITC-VAD-FMK) obtained from Millipore Corp. (Burlington, MA, USA), following previously reported methods [22]. Ca9-22 cells were cultured in 6-well plates at a density of 3 × 105 cells/well. When the cells reached 80% confluence, they were treated with various concentrations of cisplatin, with or without supplementation of 2 or 5 µM of PAC, for 24 h at 37 °C. Subsequently, the cells were trypsinized, counted, and incubated with 1 μL of FITC-VAD-FMK for 1 h at 37 °C. Flow cytometry was then performed using the FL1 channel to analyze the cells. The experiment was repeated three times to ensure consistency and reliability of the results.

### 2.7. Autophagy Assay

ICT’s (ImmunoChemistry Technologies) Autophagy assay (Red from ImmunoChemistry Technologies, Burlington, ON, Canada) was used to determine the in vitro effect of PAC (2.5 and 5 μM) and cisplatin (0 and 0.5 to 0.8 nM) on Ca9-22 cells autophagy. Following 24 h of treatment with cisplatin alone or in combination with PAC, the Ca9-22 cells were detached and incubated with an autophagy solution (diluted 1:50) for 60 min in the absence of light. Subsequently, the cells were washed three times with a cellular assay buffer and resuspended in 0.5 mL of the same buffer. The stained cells were then analyzed using flow cytometry equipped with a green/yellow laser. The Autophagy Probe, which emits at a wavelength of 590 nm and is excited at 620 nm, was used. The utilized flow cytometer was an LSRII cytometry system equipped [19,22,23]. The experiment was conducted in triplicate.

### 2.8. Measurement of ROS Levels

ROS levels were determined by flow cytometry using a specific assay kit from ImmunoChemistry Technologies, as described by Semlali et al. (2021) [19]. Briefly, Ca9-22 cells at 40 to 60% of confluence were seeded and cultured for 24 h, then exposed to different concentrations (2.5 µM and 5 µM) of PAC and (0 and 0.5 to 0.8 nM) cisplatin for 24 h. Total ROS Green dye molecule was added to 10^6^ cells/mL of each culture condition for 1 h in the dark at 37 °C before analyzing with BD Accuri C6 Plus Flow Cytometry System (BD LSRII, BD Bioscience, Mississauga, ON, Canada).

### 2.9. MitoSox Assay

As described by our previous studies [19,22], the mitochondrial oxidative stress assay was performed using MitoSOX Red dye (Invitrogen, Burlington, ON, Canada), which was detectable by flow cytometry. Briefly, Ca9-22 cells were treated with single drugs or by a combination of cisplatin and PAC for 24 h. After the treatment, 5 µM of MitoSOX™ Red was added to each sample (10^6^ cells/mL) and incubated in the dark 1 h at 37 °C. After incubation, the cells were washed twice with a warm buffer before analyzing by flow cytometry at 510 nm using LSRII cytometry from BD Bioscience.

### 2.10. Mitochondrial Membrane Potential (ΔΨm) Assay

Oral cancer cells were exposed to various concentrations of cisplatin (0, 0.5, and 0.8 nM) with or without PAC (2.5 and 5 µM) for 24 h. After trypsinization, 10^5^ cells with the fresh medium were mixed with 5 µL of DiOC_6_(3) for 30 min. Cells were washed three times with PBS and analyzed by flow cytometry analysis using a cytometer from BD Bioscience, as we previously reported [19,22].

### 2.11. Western Blotting

As described by Semlali et al. [22], gingival cancer cells were treated with PAC (2.5 and 5 μM) and cisplatin (0 and 0.5 to 0.8 nM) for 24 h. Total proteins were extracted from each condition using a lysis buffer supplemented with protease and phosphatase inhibitors. The protein concentration was determined, and Western blot analyses were performed. The proteins were denatured, separated by SDS-PAGE, and transferred onto a PVDF membrane. The membrane was blocked and incubated with primary antibodies overnight, followed by secondary antibodies, anti-mouse (554002) or anti-rabbit (554021), from BD Pharmingen (Mississauga, ON, Canada). After washing, the membrane was subjected to detection using an ECL substrate and visualized with a Versa Doc™ MP 5000 system. The entire procedure was repeated three times to ensure reproducibility and consistency of the results.

### 2.12. Wound Healing Assay

Cells (2 × 10^5^) were seeded in 12-well cell culture plates and cultured until confluence. At this step, the monolayer cultures were scratched using a sterile 200 µL pipette tip, washed with a medium, and then exposed to different concentrations of cisplatin (0, 0.5, and 0.8 nM) with or without PAC (2.5 and 5 µM). The image acquisition was taken immediately and after 6 and 24 h after drug treatment. A camera captured the pictures of each culture cell. Analysis of the open wound area was measured with the calculation of wound area (percentage) before and after 6 h and 24 h of exposure to the drugs.

### 2.13. Statistical Analysis

The statistical analysis was performed by One-way *ANOVA* and an unpaired student’s *t*-test, when required. A Bonferroni–Dunn post hoc analysis was also used when appropriate. *p* < 0.05 was considered significant between untreated cells and treated cells with PAC, cisplatin, or a combination of the two drugs.

## 3. Results

### 3.1. PAC Potentiates Cisplatin Effect on Inhibition of Oral Cancer Cell Proliferation

**Figure 1:**
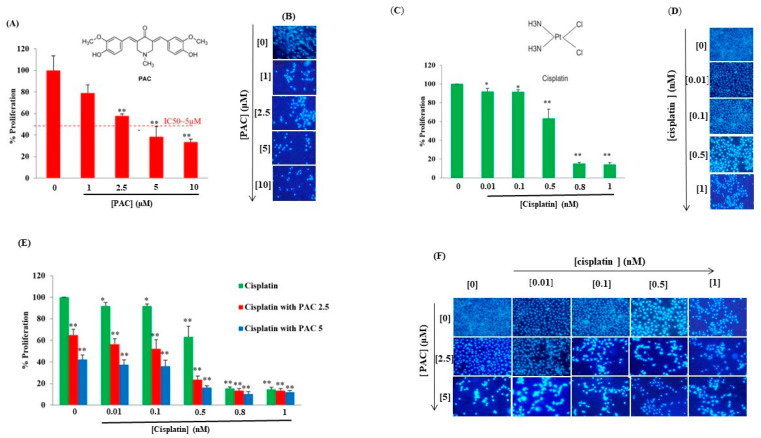
PAC potentiates cisplatin effect on inhibition of proliferation: (**A**) PAC inhibits cancer cell proliferation performed by MTT assay. Results were reported with averages of % proliferation and are considered significant when * *p* < 0.05, and ** *p* < 0.005 (*n* = 6) (**B**) Hoechst (*n* = 3). (**C**) Effect of cisplatin oral cancer proliferation (*n* = 5). (**D**) Hoechst staining (*n* = 3). (**E**) Combination PAC with cisplatin on oral cell proliferation by MTT assay (*n* = 6). (**F**) Hoechst (*n* = 3).

Initially, we examined the impact of PAC as a standalone treatment on oral cancer cells (Ca9-22) to evaluate its antiproliferative effect. Subsequently, we explored the combined effect of PAC with varying concentrations of cisplatin (ranging from 0.01 to 1 nM) using the MTT assay. As depicted in Figure 1A, the response of Ca9-22 cells to PAC exhibited a noticeable dose-dependent pattern. The IC50 for PAC treatment was determined to be approximately 5 µM. An inhibition of 21.01% ± 4.9% (*p* = 0.03), 42.325% ± 1.977% (*p* < 0.005), 61.65% ± 0.272% (*p* < 0.0005), and 66.74% ± 2.69% (*p* < 0.0005), respectively, with PAC concentrations of 1, 2.5, 5, and 10 μM was observed (Figure 1A). These results were confirmed by the Hoechst marking, where a decrease in the number of nuclei was observed gradually while increasing the concentration of PAC (Figure 1B).

Like the PAC, cisplatin alone at (0.01, 0.1, 0.5, 0.8, and 1 nM) decreases the % of proliferation of oral cancer cells in a dose-dependent manner with an IC50 = 0.7 nM. The % of inhibition decreases by 8.90% (*p* < 0.05), 8.22% (*p* < 0.05), 36.7539% (*p* < 0.005), 84.754% (*p* <0.0005), and 85.616% (*p* < 0.0005), respectively, for cisplatin at 0.01, 0.1, 0.5, 0.8, and 1 nM (Figure 1C). These results were confirmed by the Hoechst marking, where the nucleus number gradually reduced by increasing the concentration of cisplatin (Figure 1D). A combination of cisplatin (0.01, 0.1, 0.5, 0.8, and 1 nM) and PAC (2.5 and 5 μM) was more effective in reducing cancer cell proliferation. The cisplatin IC50 in combination with 2.5 μM of PAC has become of the order of 0.2 nM, a decrease of three times. Indeed, with the concentration of PAC at 5 μM, the IC50 was about 0.07 nM, a decrease of 10 times (Figure 1C). The proliferation results were also confirmed by the Hoechst marking, where a greater decrease in the number of nuclei of Ca9-22 cells was observed and was dependent on the concentration of cisplatin and PAC (Figure 1F).

### 3.2. The Combination of PAC and Cisplatin Inhibits the Capacity of Cancer Cells to Form Colonies

**Figure 2:**
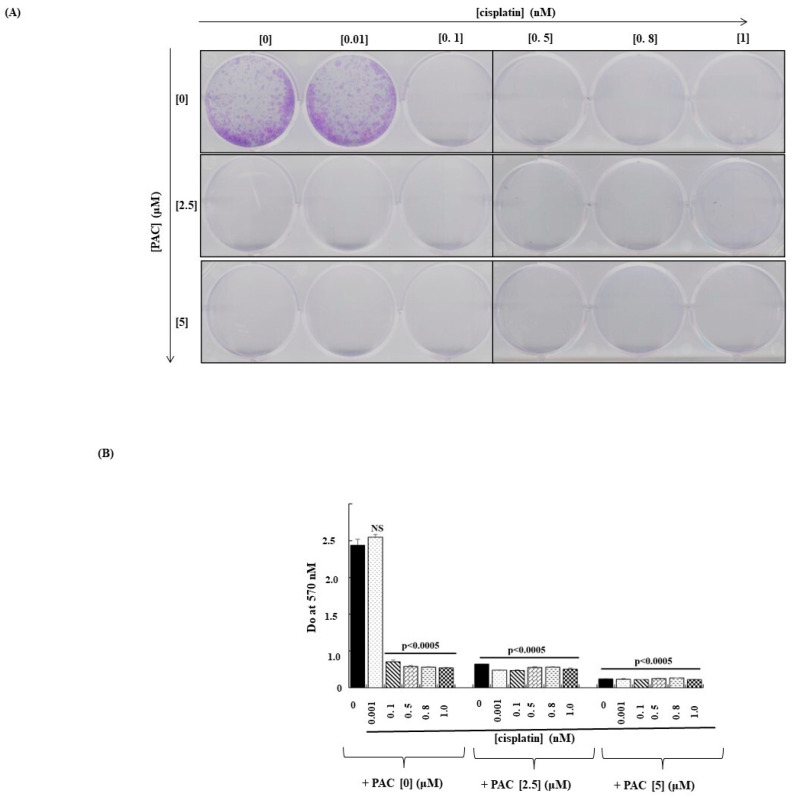
PAC potentiates the effect of cisplatin inhibitor on colony formation in oral cancer cells: (**A**) Microscopic observation of colony formation through violet crystal staining. (**B**) Quantification of the number of colonies. The results were reported by Do at 570 nM after lysis by acetic acid (*n* = 3). NS: Non- significant.

As shown in Figure 2, colony formation decreased significantly with single treatments with cisplatin and PAC. Moreover, this inhibition was more important after combining the two treatments (Figure 2A). These observations were confirmed by the crystal violet assay (Figure 2B), showing a decrease in the absorbance when the cells were exposed to cisplatin or PAC alone. A combination of both molecules led to a better decrease in colony formation by oral cancer cells.

### 3.3. PAC Potentiates the Cisplatin Effect by Inducing Apoptosis of Oral Cancer Cells

**Figure 3:**
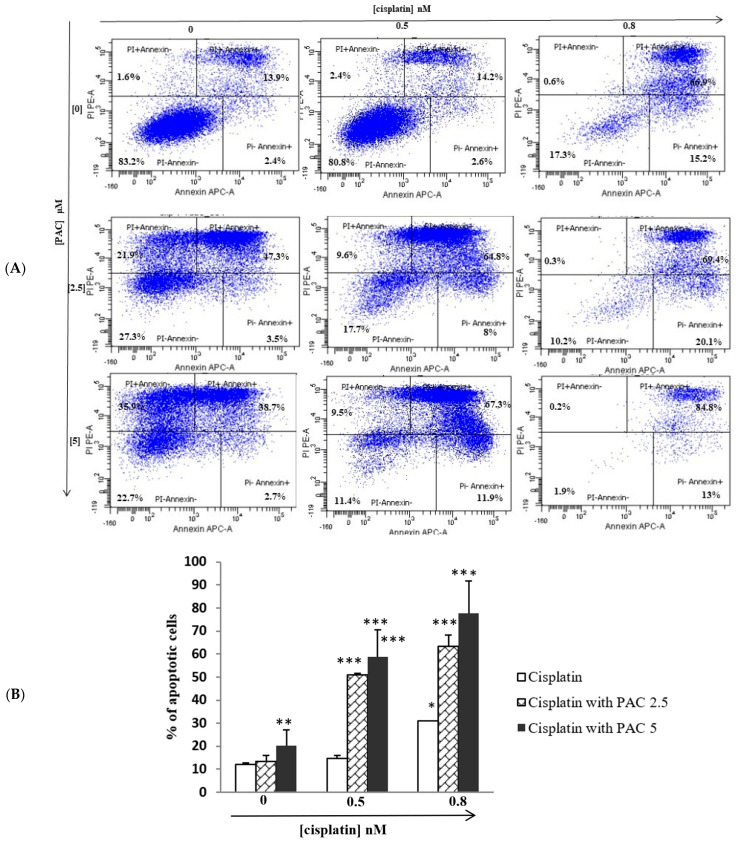
PAC potentiates the effect of cisplatin on apoptosis: Gingival squamous cell carcinoma (Ca9-22) was exposed to different concentrations of cisplatin (0.5 nM and 0.8 nM), with PAC (0, 2.5, and 5 µM), or with combination of two drugs for 24 h. (**A**) Apoptosis cells were measured using a PI/annexin kit. PI^−^ annexin^−^ represents the percentage of cells non staining by PI and annexin (Viable cells). PI^−^ annexin^+^ is the percentage of cells stained by annexin (early apoptotic cells). PI^+^ annexin^+^ (late apoptotic cells). PI^+^ annexin^−^ (dead cells). (**B**) Diagram representing the percentage of apoptotic cells from 3 independent experiments: * *p* < 0.05, ** *p* < 0.005, and *** *p* < 0.0005.

Figure 3 shows that a single treatment with PAC or cisplatin significantly induces oral cancer cell apoptosis. Combined PAC at 2.5 or 5 μM (concentration around of IC50 for PAC) with different concentrations of cisplatin (0, 0.5, and 0.8 nM) (concentration around of IC50 for cisplatin) drastically increased cell apoptosis than cells treated with cisplatin alone. These data indicate that PAC enhances cisplatin cytotoxicity on cancer cells by triggering an apoptosis process. Indeed, with cisplatin, the percentage of apoptotic cells increased from 17.9% at the control to 19.2% and 82.7%, respectively, with the concentrations of cisplatin (0.5 and 0.8 nM). On the other hand, the apoptotic cell rate increased with PAC from 17.9% to 72.7% and 77.3%, respectively, with concentrations (0, 2.5, and 5 μM). However, the percentage of the apoptotic cells increases when combining PAC with cisplatin, reaching 82.3% and 89.8%, respectively, when we combined PAC at 2.5 μM with cisplatin at 0.5 or 0.8 nM. With the PAC at 5 μM and cisplatin (0.5 and 0.8 μM), the percentage of apoptotic cells becomes, respectively, 88.6% and 98.1% (Figure 3A,B).

### 3.4. PAC Potentiates Caspases Activities Induced by Cisplatin on Oral Cancer Cells

**Figure 4:**
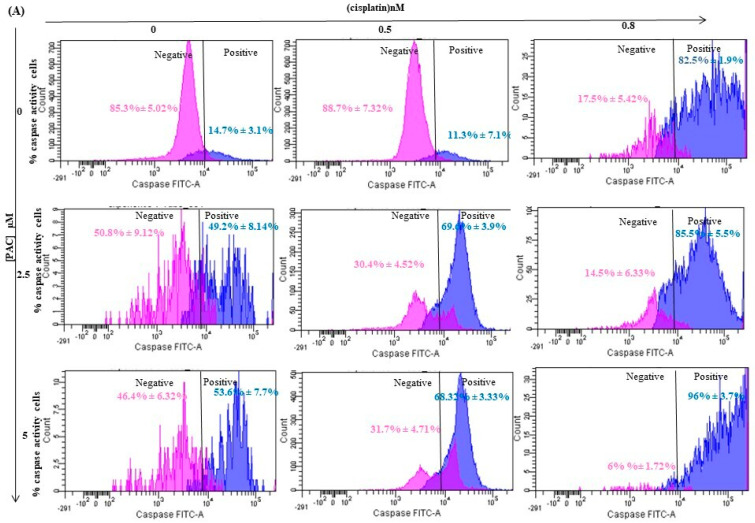

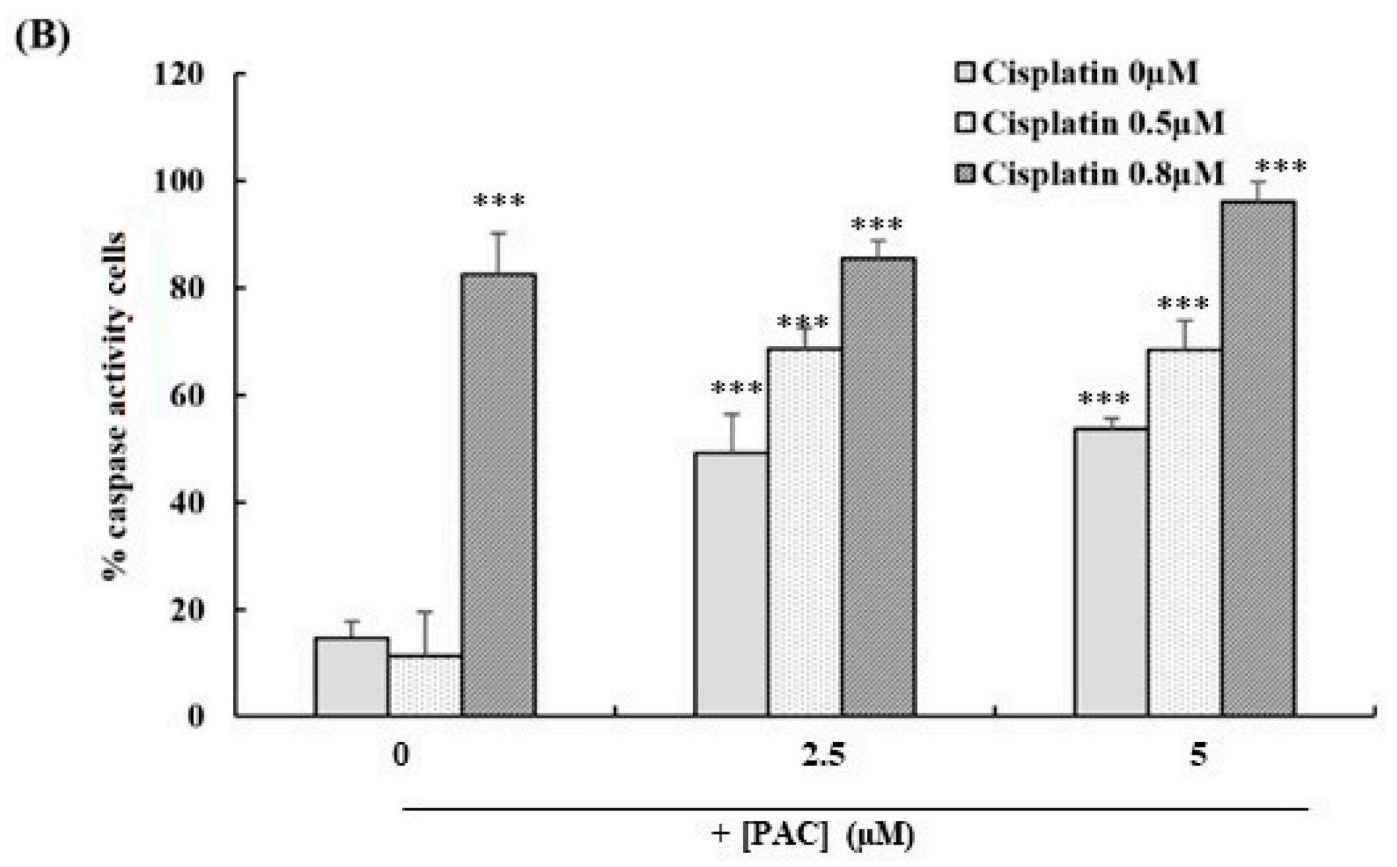
PAC potentiates the cisplatin effect by promoting the caspase activity of oral cancer cells. (**A**) Caspase activity was assessed using a caspase detection kit (TITC-VAD-FMK) and analyzed by flow cytometry using the FL1 channel (*n* = 3). (**B**) Diagram representing the percentage of apoptotic cells from 3 independent experiments. *** *p* < 0.0005 compared to untreated cells.

As shown in Figure 4, cisplatin induces apoptosis by inducing caspase activity. The percentage of cells stained by FITC-VAD-FMK increased from 14.7% in the control to 82.5% when Ca9-22 cells were stimulated with 0.8 nM of cisplatin. The addition of PAC at 2.5 µM to cisplatin treatment significantly increased caspase activity. Indeed, the percentage of caspase-positive cells increased to 49.2% with 2.5 µM of PAC compared to control cells and to 85.5% when the cells were treated with 2.5 µM of PAC + 0.8 nM of cisplatin. The caspase activity was further increased when using 5 µM of PAC. Indeed, caspase-positive cells reached 96% with 5 µM of PAC in combination with 0.8 nM of cisplatin (Figure 4).

### 3.5. Concomitant Use of PAC and Cisplatin Increased Cancer Cell Autophagy

**Figure 5:**
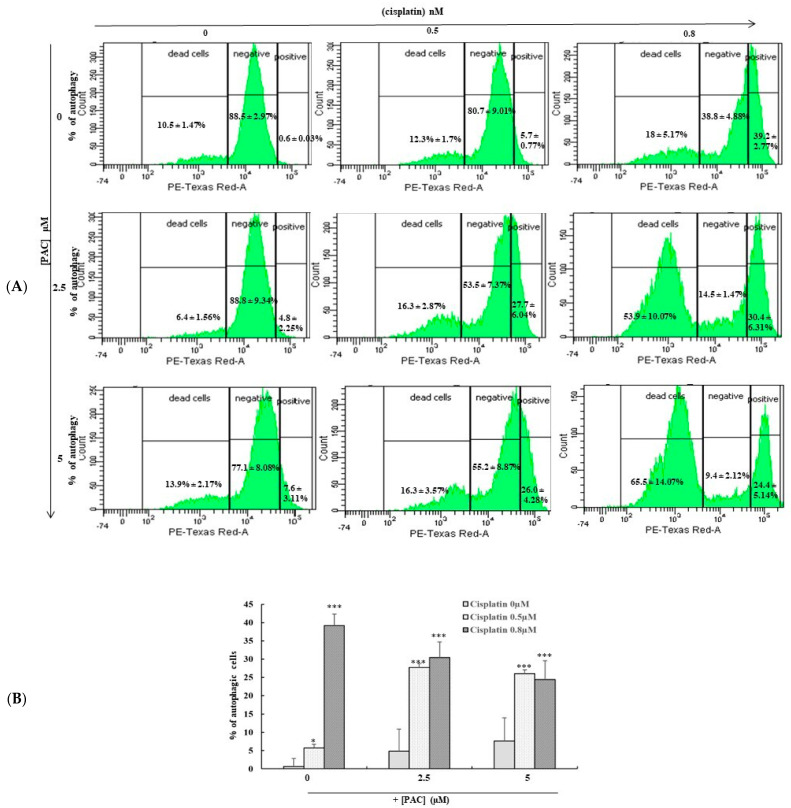
PAC potentiates the cisplatin effect by promoting autophagy of oral cancer cells: (**A**) Flow cytometry analysis after treatment by combined treatment with cisplatin and PAC or with single drugs for 24 h. The Ca9-22 cells were detached and incubated with the autophagy solution (1:50) and incubated in the dark for 1 h, before analyzing by flow cytometry using a green/yellow laser (*n* = 3). (**B**) Diagram representing the percentage of positive autophagic cells from 3 independent experiments. * *p* < 0.05 *** *p* < 0.0005 compared to untreated cells.

We then examined whether the combination of PAC and cisplatin favors the initiation of autophagy. The results in Figure 5 show that PAC promotes the autophagy of cancer cells even at low doses of cisplatin. Indeed, after 24 h of incubation with an increasing concentration of cisplatin, the percentage of autophagic cells increased considerably to 39.2% following the treatment of cells with 0.8 nm of cisplatin. Similarly, the exposure of cancer cells to PAC only at 5 µM led to cell autophagy reaching 7.6%. The combination of cisplatin and PAC increased the ratio of autophagic+/autophagic− cells in living cells. In untreated cells, the percentage of autophagic+ cells was about 0.6%; the percentage of unlabeled living cells was 88.5%; and 10.5% were dead cells. The combination of PAC (5 µM) with cisplatin (0.8 nM) significantly decreased the living-labeled cells to 9.4%, and the percentage of living autophagic+ cells was 24.4%. In contrast, a large portion of the cells were dead (65.5%).

### 3.6. PAC Potentiates Total and Mitochondrial Oxidative Stress Induced by Cisplatin in Oral Cancer Cells

**Figure 6:**
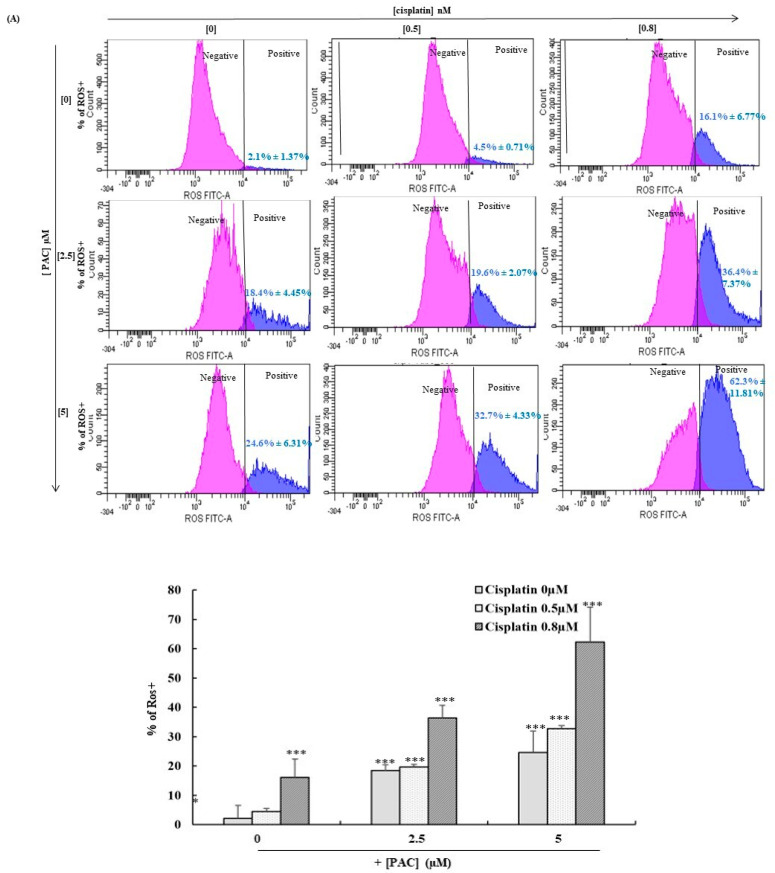

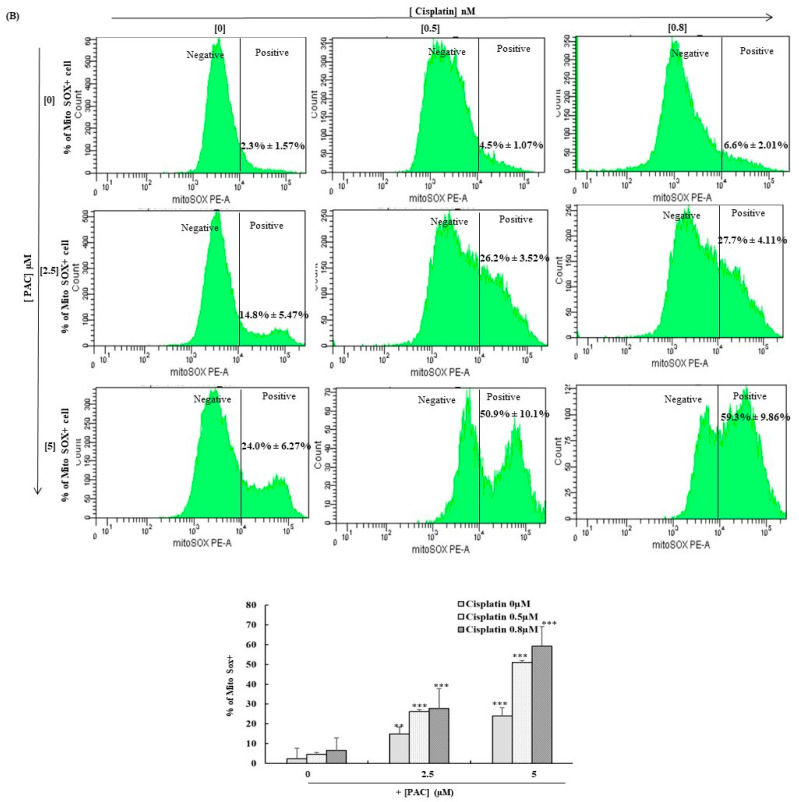
PAC promotes the effect of cisplatin on the inhibition of oxidative stress in oral cancer cells. (**A**) The quantification of ROS in flow cytometry (*n* = 4) showed inhibition when the Ca9-22 cells were treated with PAC and cisplatin for 24 h. The pink peak represents the % of ROS^+^ cells, and the blue peak is the percentage of ROS^−^ cells. (**B**) MitoSox expression performed by flow cytometry (*n* = 4). ** *p* < 0.005 *** *p* < 0.0005 compared to untreated cells.

Firstly, we investigated the effect of a combination of PAC and cisplatin on the total intracellular ROS using flow cytometry. In this step, the cells were at 40 to 60% of their maximum confluence because the ROS is confluence-dependent. As shown in Figure 6A, cisplatin significantly increased the ROS expression in a dose-dependent manner from 2.1% in untreated cells to 16.1% when the cells were stimulated by 0.8 nM of cisplatin. Additionally, PAC increased the expression of ROS dose-dependently, from 2.1% in untreated cells to 24.6% when the cells were stimulated with 5 µM of PAC. The exposure of the cells to PAC (5 µM) and cisplatin (0.8 nM) at the same time increased the ROS expression to reach 62.3%.

In the second part, we investigated the effect of a combination of cisplatin and PAC on mitochondrial ROS expression using the MitoSOX probe. Our data show (Figure 6B) that the single treatment with PAC and cisplatin increased MitoSOX from 2.3% in untreated cells to 6.6% for cisplatin-treated Ca9-22 cells at 0.8 nM, and 24% for PAC-treated cells at 5 µM. However, the percentage of MitoSOX-positive cells considerably increased to reach a value of 59.9% with a combined treatment of 0.8 nM of cisplatin and 5 µM of PAC (Figure 6B).

### 3.7. Combined PAC with Cisplatin Drastically Enhances the Expression of Mitochondrial Membrane Potential (ΔΨm)

**Figure 7:**
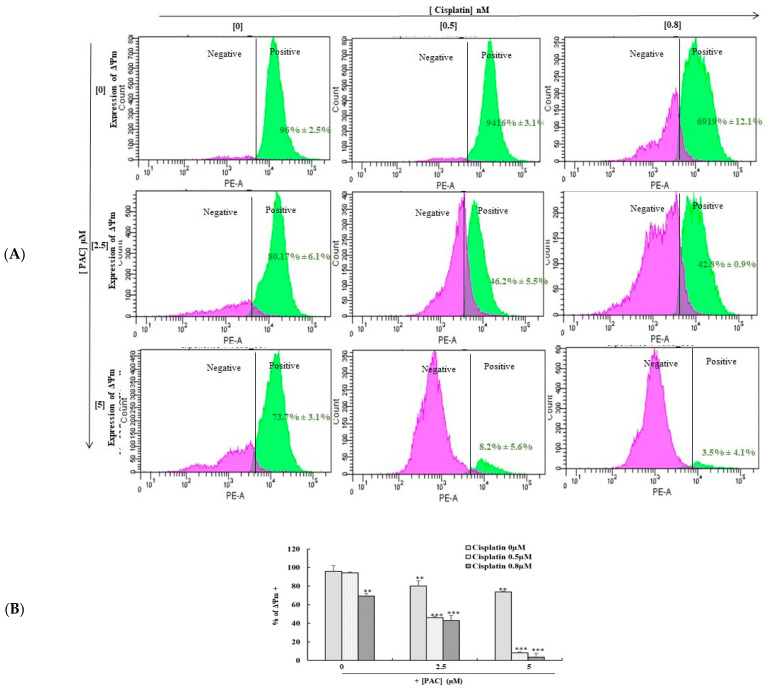
PAC promotes the effect of cisplatin on the inhibition of mitochondrial membrane potential. (**A**). (ΔΨm) Expression was measured by DiOC6(3) using flow cytometry. The pink peak represents the % of cells ΔΨm^+^ cells, and the blue peak is the percentage of ΔΨm^−^ cells (*n* = 3). ** *p* < 0.005 *** *p* < 0.0005 compared to untreated cells. (**B**) Diagram representing the percentage of positive ΔΨm cells from 3 independent experiments. ** *p* < 0.05 *** *p* < 0.0005 compared to untreated cells.

Combining PAC with cisplatin drastically enhances the expression of mitochondrial membrane potential (ΔΨm). The mitochondrial membrane potential (ΔΨm) is an important indicator of mitochondrial activity because of its role in the ATP production process. Data in Figure 7 show that combined cisplatin and PAC further induce the mitochondrial membrane potential (ΔΨm) alterations compared to the cells treated with PAC alone or cisplatin alone. Indeed, the ΔΨm decreased from 99.75% in untreated cells to 3.5% in cells treated simultaneously with 5 µM of PAC and 0.8 nm of cisplatin (Figure 7). However, cisplatin (0.8 nM) treatment led to 69.9% of positive cells, and PAC (5 µM) treatment showed 73.7% positive cells after 24 h of exposure to PAC, cisplatin, or combined molecules. These data confirm our proliferation results showing that the concomitant use of cisplatin and PAC highly inhibits cancer cell proliferation compared to treatment with each molecule alone.

### 3.8. PAC and Cisplatin Inhibit Oral Cancer Cell Migration

**Figure 8:**
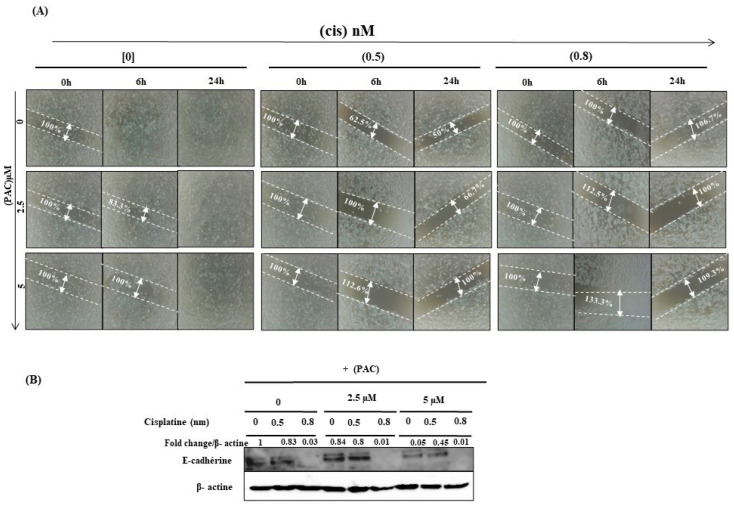
The synergistic effect of PAC with cisplatin promotes further inhibition of oral cell migration. (**A**) Illustrates the results of the wound healing assay (*n* = 3). (**B**) Represents Western blotting for the E-cadherin protein (*n* = 3).

Our in vitro results demonstrated that PAC has proven itself as a kind of complementary effective drug for oral chemotherapy. In addition, a combined sub-lethal concentration of PAC with cisplatin dramatically inhibits oral cell migration compared to the cells when it’s treated with a single treatment of cisplatin or PAC (Figure 8A). In addition, PAC decreases the epithelial-to-mesenchymal transition (EMT) gene such as the E-cadherin gene (Figure 8B).

## 4. Discussion

Cisplatin is the oldest and most often used chemotherapeutic agent in various solid cancer therapies [24]. Since its synthesis and discovery as a DNA intercalating agent known for its anti-tumor activity, cisplatin has gained momentum in the chemotherapy of many types of cancer, including oral cancer. However, its therapeutic success presents many limitations linked to resistance acquisition, and it induces disease relapse in 80% of cancer cases [25]. The scientific community is now leaning toward developing a complementary alternative/complementary drug to improve cisplatin’s bioactivity and reduce its adverse effects. In the current study, we evaluated the combined effects of PAC and cisplatin on oral cancer therapy. Our in vitro results demonstrated that PAC was proven to be a complementary, effective drug for oral chemotherapy with fewer side effects. In addition, the simultaneous combination of cisplatin with a sub-lethal concentration of PAC dramatically potentiated the cell-killing efficacy of cisplatin through a greater induction of DNA double-strand breaks. Furthermore, the combination of PAC and cisplatin improved the toxicity of cisplatin against cancer cells via a greater induction of cell apoptosis, autophagy, and oxidative stress effects. It was reported that PAC had been extensively used in research to treat several cancers [16,17,18]. Recently, PAC was shown to exhibit powerful anti-oral cancer features by inducing apoptosis, autophagy, and oxidative stress of the tumor oral cells by involving various signaling pathways such as MAPK, Wnt, NF-kappa B, and caspases. This effect is marginal on normal gingival cells [19]. This indicates that PAC could be a powerful therapeutic agent to be used as an alternative or complementary treatment to chemotherapy for oral cancer for several reasons. Firstly, the combination of PAC and cisplatin presents a strong synergistic chemotherapeutic effect on a great proportion of oral cancer cells. Secondly, cancer cells treated with PAC and cisplatin were significantly less invasive and had a lower migratory capacity than those treated with a single drug. Thirdly, PAC combined with cisplatin potentializes oral cancer cells’ autophagy and enhances oxidative stress. Fourth, this combination can inhibit many cancer pathways, such as ERK1/2, NF-kB, STATs, and p38. Our results were in concordance with our recent data using anethole as adjuvant treatment with a synergic effect with cisplatin on oral cancer therapy by inhibiting MAPKase, beta-catenin, and NF-κB pathways [26].

These data show that PAC and cisplatin exhibit powerful anti-oral cancer activity via two different pathways. Cisplatin exerts its antitumor effects through its ability to induce DNA damage. PAC is a powerful antiproliferative agent that can disrupt cell cycle progression and induce oral cancer cell apoptosis through caspase 3 and caspase 9 and by inducing oral cancer cell autophagy [16,17,18,19]. It was largely reported that combined curcumin and cisplatin suppressed colony formation and cell proliferation, particularly in lung cancer cells [27]. Another finding has highlighted that this combination could suppress JAK/STAT3 signaling pathways involved with papillary thyroid cancer cell growth and proliferation and may provide better therapeutic outcomes [28]. Similar research demonstrated that co-treatment with curcumin and cisplatin increased bladder cancer cells’ apoptosis compared to cells when treated with a single drug. In this study, the authors found that the co-treatment of cisplatin and curcumin caused an increase in the p53 and p21 gene expression [27,29,30]. Other in vivo and in vitro studies demonstrated that combined treatments of cisplatin and curcumin inhibited tumor angiogenesis in head and neck cancer cells [31]. However, Saghatelyan et al., using a randomized double-blind placebo-controlled clinical study, assessed the efficacy and safety of the combination of curcumin and chemotherapy among women with advanced and metastatic breast cancer [32]. Other natural products, such as ginger, were cited in the literature as complementary agents to chemotherapy. A recent study by Famurewa et al. (2020) indicated that cisplatin combined with fresh ginger juice mitigates cisplatin testicular damage considerably by abrogating oxidative stress and anti-inflammatory mechanisms [33]. A similar observation was found when certain cancer cells were sensitized to treatment combined with cisplatin with the same flavonoids in both ovarian and prostate cancer cells [34,35]. Recently, many studies have clearly indicated that quercetin inhibits cisplatin’s nephrotoxicity by ameliorating tubular damage [36]. All these authors concluded that the potentiation of cisplatin’s effects against cancer cells is now possible by natural molecules and their derivates, which can enhance the efficiency and reduce the side effects of this potent anticancer molecule.

Targeting cell death is the most effective chemotherapeutic strategy. Our study also clearly demonstrated that PAC and cisplatin arrested oral cancer cell proliferation by destabilizing cell-cycle distributions, downregulating the expression of cyclin D1, and upregulating of cyclin-dependent kinase inhibitors, such as the p21 gene, as observed in our previous work with the single treatment with PAC [19]. Clinically, targeting intrinsic and extrinsic apoptosis pathways is a key mechanism of chemotherapeutic drugs that are able to control tumor progression [37,38], and constitutes one of the most important therapies promoting the effective elimination of cancer cells. This cell death process is often mediated by multiple factors, including total and mitochondrial cellular stress. Oxidative stress has also been proven to play a key role in the pathogenesis and progression of cancers [39].

Targeting the stimulation of autophagy cells can be achieved essentially through cellular stress and by the inhibition of the mTOR pathway (ref). For a decade, the role of autophagy in cancer therapy has been controversial (to prevent or promote) and has been hotly debated among scientists. Few studies suggest that the induction of autophagy may prevent cancer progression [40]. Conversely, many other studies have suggested that autophagy is required for optimal anticancer immunosurveillance and has been proposed as a potential therapeutic strategy in cancer [41].

Targeting the induction of ROS has been reported as a potential chemotherapeutic drug for multiple cancers [42]. It has been found that WZ35, a novel curcumin analog, synergistically enhanced the anti-gastric cancer activity of cisplatin by increasing the ROS level [43]. The synergistic effect of cisplatin and PAC on the inhibition of the membrane’s mitochondrial potential ΔΨm was closely linked to increased ROS levels in mitochondria in accordance with cytotoxicity results, indicating an impaired functional capacity of the mitochondria and strong tumorigenicity impairment caused by a combination of cisplatin and PAC. Many studies have reported that a loss of the mitochondrial membrane potential is often associated with ROS-mediated apoptosis and cytotoxicity [44,45].

## 5. Conclusions

We have demonstrated that PAC, a new curcumin analog, has significant potential to protect against cisplatin-induced toxicity in oral cancer cells. Moreover, a combination therapy of cisplatin and PAC has been shown to be effective in alleviating resistance to cisplatin treatment. Therefore, the application of cisplatin- and PAC-based drug formulations could be a novel therapeutic strategy to combat human oral cancers.

## References

[1] Galluzzi L., Senovilla L., Vitale I., Michels J., Martins I., Kepp O., Castedo M., Kroemer G. (2012). Molecular mechanisms of cisplatin resistance. Oncogene.

[2] Klaunig J.E. (2018). Oxidative Stress and Cancer. Curr. Pharm. Des..

[3] Kamath S.S. (2014). Risk Factors Assessment of the Difficult Intubation Using Intubation Difficulty Scale (IDS). J. Clin. Diagn. Res..

[4] Ghosh S. (2019). Cisplatin: The first metal based anticancer drug. Bioorg. Chem..

[5] Yao X., Panichpisal K., Kurtzman N., Nugent K. (2007). Cisplatin nephrotoxicity: A review. Am. J. Med. Sci..

[6] Furness S., Glenny A.-M., Worthington H.V., Pavitt S., Oliver R., Clarkson J.E., Macluskey M., Chan K.K., Conway D.I. (2011). Interventions for the treatment of oral cavity and oropharyngeal cancer: Chemotherapy. Cochrane Database Syst. Rev..

[7] Silverman S. (1999). Oral cancer: Complications of therapy. Oral Surg. Oral Med. Oral Pathol. Oral Radiol. Endodontol..

[8] Brami C., Bao T., Deng G. (2016). Natural products and complementary therapies for chemotherapy-induced peripheral neuropathy: A systematic review. Crit. Rev. Oncol..

[9] Mitra S., Dash R. (2018). Natural Products for the Management and Prevention of Breast Cancer. Evid.-Based Complement. Altern. Med..

[10] Newman D.J., Cragg G.M. (2016). Natural products as sources of new drugs from 1981 to 2014. J. Nat. Prod..

[11] Islam S.S., Al-Sharif I., Sultan A., Al-Mazrou A., Remmal A., Aboussekhra A. (2018). Eugenol potentiates cisplatin anti-cancer activity through inhibition of ALDH-positive breast cancer stem cells and the NF-κB signaling pathway. Mol. Carcinog..

[12] Li X., Guo S., Xiong X.-K., Peng B.-Y., Huang J.-M., Chen M.-F., Wang F.-Y., Wang J.-N. (2019). Combination of quercetin and cisplatin enhances apoptosis in OSCC cells by downregulating xIAP through the NF-κB pathway. J. Cancer.

[13] Maheshwari R.K., Singh A.K., Gaddipati J., Srimal R.C. (2006). Multiple biological activities of curcumin: A short review. Life Sci..

[14] Adams B.K., Ferstl E.M., Davis M.C., Herold M., Kurtkaya S., Camalier R.F., Hollingshead M.G., Kaur G., Sausville E.A., Rickles F.R. (2004). Synthesis and biological evaluation of novel curcumin analogs as anti-cancer and anti-angiogenesis agents. Bioorg. Med. Chem..

[15] Selvendiran K., Ahmed S., Dayton A., Ravi Y., Kuppusamy M.L., Bratasz A., Rivera B.K., Kálai T., Hideg K., Kuppusamy P. (2010). HO-3867, a Synthetic Compound, Inhibits the Migration and Invasion of Ovarian Carcinoma Cells through Downregulation of Fatty Acid Synthase and Focal Adhesion Kinase. Mol. Cancer Res..

[16] Al-Howail H.A., Hakami H.A., Al-Otaibi B., Al-Mazrou A., Daghestani M.H., Al-Jammaz I., Al-Khalaf H.H., Aboussekhra A. (2016). PAC down-regulates estrogen receptor alpha and suppresses epithelial-to-mesenchymal transition in breast cancer cells. BMC Cancer.

[17] Al-Hujaily E.M., Mohamed A.G., Al-Sharif I., Youssef K.M., Manogaran P.S., Al-Otaibi B., Al-Haza’a A., Al-Jammaz I., Al-Hussein K., Aboussekhra A. (2011). PAC, a novel curcumin analogue, has anti-breast cancer properties with higher efficiency on ER-negative cells. Breast Cancer Res. Treat..

[18] Al-Qasem A., Al-Howail H.A., Al-Swailem M., Al-Mazrou A., Al-Otaibi B., Al-Jammaz I., Al-Khalaf H.H., Aboussekhra A. (2015). PAC exhibits potent anti-colon cancer properties through targeting cyclin D1 and suppressing epithelial-to-mesenchymal transition. Mol. Carcinog..

[19] Semlali A., Contant C., Al-Otaibi B., Al-Jammaz I., Chandad F. (2021). The curcumin analog (PAC) suppressed cell survival and induced apoptosis and autophagy in oral cancer cells. Sci. Rep..

[20] Fuchs Y., Steller H. (2015). Live to die another way: Modes of programmed cell death and the signals emanating from dying cells. Nat. Rev. Mol. Cell Biol..

[21] Long J.S., Ryan K.M. (2012). New frontiers in promoting tumour cell death: Targeting apoptosis, necroptosis and autophagy. Oncogene.

[22] Semlali A., Beji S., Ajala I., Rouabhia M. (2021). Effects of tetrahydrocannabinols on human oral cancer cell proliferation, apoptosis, autophagy, oxidative stress, and DNA damage. Arch. Oral Biol..

[23] Contant C., Rouabhia M., Loubaki L., Chandad F., Semlali A. (2021). Anethole induces anti-oral cancer activity by triggering apoptosis, autophagy and oxidative stress and by modulation of multiple signaling pathways. Sci. Rep..

[24] Ali R., Aouida M., Sulaiman A.A., Madhusudan S., Ramotar D. (2022). Can Cisplatin Therapy Be Improved? Pathways That Can Be Targeted. Int. J. Mol. Sci..

[25] Ai Z., Lu Y., Qiu S., Fan Z. (2016). Overcoming cisplatin resistance of ovarian cancer cells by targeting HIF-1-regulated cancer metabolism. Cancer Lett..

[26] Semlali A., Ajala I., Beji S., Al-Zharani M.M., Rouabhia M. (2023). Synergistic effect of anethole and Platinum Drugs cisplatin against oral cancer cell growth and migration by inhibiting MAPKase, beta catenin and NF-κB pathways. Pharmaceuticals.

[27] Baharuddin P., Satar N., Fakiruddin K.S., Zakaria N., Lim M.N., Yusoff N.M., Zakaria Z., Yahaya B.H. (2016). Curcumin improves the efficacy of cisplatin by targeting cancer stem-like cells through p21 and cyclin D1-mediated tumour cell inhibition in non-small cell lung cancer cell lines. Oncol. Rep..

[28] Khan A.Q., Ahmed E.I., Elareer N., Fathima H., Prabhu K.S., Siveen K.S., Kulinski M., Azizi F., Dermime S., Ahmad A. (2020). Curcumin-Mediated Apoptotic Cell Death in Papillary Thyroid Cancer and Cancer Stem-Like Cells through Targeting of the JAK/STAT3 Signaling Pathway. Int. J. Mol. Sci..

[29] Park B.H., Lim J.E., Jeon H.G., Seo S.I., Lee H.M., Choi H.Y., Jeon S.S., Jeong B.C. (2016). Curcumin potentiates antitumor activity of cisplatin in bladder cancer cell lines via ROS-mediated activation of ERK1/2. Oncotarget.

[30] Rutz J., Janicova A., Woidacki K., Chun F.K.-H., Blaheta R.A., Relja B. (2020). Curcumin—A Viable Agent for Better Bladder Cancer Treatment. Int. J. Mol. Sci..

[31] Kumar B., Yadav A., Hideg K., Kuppusamy P., Teknos T.N., Kumar P. (2014). A Novel Curcumin Analog (H-4073) Enhances the Therapeutic Efficacy of Cisplatin Treatment in Head and Neck Cancer. PLoS ONE.

[32] Saghatelyan T., Tananyan A., Janoyan N., Tadevosyan A., Petrosyan H., Hovhannisyan A., Hayrapetyan L., Arustamyan M., Arnhold J., Rotmann A.-R. (2020). Efficacy and safety of curcumin in combination with paclitaxel in patients with advanced, metastatic breast cancer: A comparative, randomized, double-blind, placebo-controlled clinical trial. Phytomedicine.

[33] Famurewa A.C., Ekeleme-Egedigwe C.A., Onwe C.S., Egedigwe U.O., Okoro C.O., Egedigwe U.J., Asogwa N.T. (2020). Ginger juice prevents cisplatin-induced oxidative stress, endocrine imbalance and NO/iNOS/NF-κB signalling via modulating testicular redox-inflammatory mechanism in rats. Andrologia.

[34] Erdogan S., Turkekul K., Serttas R., Erdogan Z. (2017). The natural flavonoid apigenin sensitizes human CD44(+) prostate cancer stem cells to cisplatin therapy. Biomed. Pharmacother..

[35] Li J., Wang Y., Lei J.-C., Hao Y., Yang Y., Yang C.-X., Yu J.-Q. (2014). Sensitisation of ovarian cancer cells to cisplatin by flavonoids from *Scutellaria barbata*. Nat. Prod. Res..

[36] Casanova A.G., Prieto M., Colino C.I., Gutiérrez-Millán C., Ruszkowska-Ciastek B., de Paz E., Martín Á., Morales A.I., López-Hernández F.J. (2021). A Micellar Formulation of Quercetin Prevents Cisplatin Nephrotoxicity. Int. J. Mol. Sci..

[37] Carneiro B.A., El-Deiry W.S. (2020). Targeting apoptosis in cancer therapy. Nat. Rev. Clin. Oncol..

[38] Tang C., Zhao C.-C., Yi H., Geng Z.-J., Wu X.-Y., Zhang Y., Liu Y., Fan G. (2020). Traditional Tibetan Medicine in Cancer Therapy by Targeting Apoptosis Pathways. Front. Pharmacol..

[39] Su Z., Yang Z., Xu Y., Chen Y., Yu Q. (2015). Apoptosis, autophagy, necroptosis, and cancer metastasis. Mol. Cancer.

[40] Galluzzi L., Pietrocola F., Bravo-San Pedro J.M., Amaravadi R.K., Baehrecke E.H., Cecconi F., Codogno P., Debnath J., Gewirtz D.A., Karantza V. (2015). Autophagy in malignant transformation and cancer progression. EMBO J..

[41] Levy J.M.M., Thorburn A. (2011). Targeting autophagy during cancer therapy to improve clinical outcomes. Pharmacol. Ther..

[42] Zaidieh T., Smith J.R., Ball K.E., An Q. (2019). ROS as a novel indicator to predict anticancer drug efficacy. BMC Cancer.

[43] He W., Xia Y., Cao P., Hong L., Zhang T., Shen X., Zheng P., Shen H., Liang G., Zou P. (2019). Curcuminoid WZ35 synergize with cisplatin by inducing ROS production and inhibiting TrxR1 activity in gastric cancer cells. J. Exp. Clin. Cancer Res..

[44] Ye T., Zhu S., Zhu Y., Feng Q., He B., Xiong Y., Zhao L., Zhang Y., Yu L., Yang L. (2016). Cryptotanshinone induces melanoma cancer cells apoptosis via ROS-mitochondrial apoptotic pathway and impairs cell migration and invasion. Biomed. Pharmacother..

[45] Zhou Y.-J., Zhang S.-P., Liu C.-W., Cai Y.-Q. (2009). The protection of selenium on ROS mediated-apoptosis by mitochondria dysfunction in cadmium-induced LLC-PK1 cells. Toxicol. Vitr..

